# Protein Aggregation Formed by Recombinant cp19k Homologue of *Balanus albicostatus* Combined with an 18 kDa N-Terminus Encoded by pET-32a(+) Plasmid Having Adhesion Strength Comparable to Several Commercial Glues

**DOI:** 10.1371/journal.pone.0136493

**Published:** 2015-08-28

**Authors:** Chao Liang, Yunqiu Li, Zhiming Liu, Wenjian Wu, Biru Hu

**Affiliations:** 1 Department of Chemistry and Biology, College of Science, National University of Defense Technology, Changsha, Hunan, China; 2 State Key Laboratory of NBC Protection for Civilian, Beijing, China; Duke University Marine Laboratory, UNITED STATES

## Abstract

The barnacle is well known for its tenacious and permanent attachment to a wide variety of underwater substrates, which is accomplished by synthesizing, secreting and curing a mixture of adhesive proteins termed “barnacle cement”. In order to evaluate interfacial adhesion abilities of barnacle cement proteins, the *cp19k* homologous gene in *Balanus albicostatus* (*Balcp19k*) was cloned and expressed in *Escherichia coli*. Here, we report an intriguing discovery of a gel-like super adhesive aggregation produced by Trx-Balcp19k, a recombinant Balcp19k fusion protein. The Trx-Balcp19k consists of an 18 kDa fragment at the N-terminus, which is encoded by pET-32a(+) plasmid and mainly comprised of a thioredoxin (Trx) tag, and Balcp19k at the C-terminus. The sticky aggregation was designated as “Trx-Balcp19k gel”, and the bulk adhesion strength, biochemical composition, as well as formation conditions were all carefully investigated. The Trx-Balcp19k gel exhibited strong adhesion strength of 2.10 ± 0.67 MPa, which was approximately fifty folds higher than that of the disaggregated Trx-Balcp19k (40 ± 8 kPa) and rivaled those of commercial polyvinyl acetate (PVA) craft glue (Mont Marte, Australia) and UHU glue (UHU GmbH & Co. KG, Germany). Lipids were absent from the Trx-Balcp19k gel and only a trace amount of carbohydrates was detected. We postulate that the electrostatic interactions play a key role in the formation of Trx-Balcp19k gel, by mediating self-aggregation of Trx-Balcp19k based on its asymmetric distribution pattern of charged amino acids. Taken together, we believe that our discovery not only presents a promising biological adhesive with potential applications in both biomedical and technical fields, but also provides valuable paradigms for molecular design of bio-inspired peptide- or protein-based materials.

## Introduction

During the long history of evolution, numerous marine invertebrates, such as echinoderm sea stars [1], mollusc mussels [2], arthropod barnacles [3], annelid tubeworms [4, 5], and so on, have attained the unique capability to temporarily or permanently adhere to underwater substrates through producing, secreting and solidifying waterborne multi-protein adhesives. Given the not yet satisfied demand of strong underwater adhesives in both biomedical and technical fields, on one hand, marine adhesive proteins provide ideal models for design of novel bio-inspired underwater adhesives or coatings [6–10], due to their excellent adhesion ability and special waterproof property. On the other hand, the undesired gregarious attachment of sessile marine invertebrates, including barnacles and mussels on submerged manmade structures usually leads to substantial environmental damages and economical losses [11–13]. Thus, to reveal the complicated underwater adhesion mechanisms of barnacles and mussels will not only advance the research of biomimetic underwater adhesives or coatings, but also speed the development of novel environmentally friendly antifouling technologies [12–15].

Being among the most notorious marine fouling organisms, barnacles rely on proteinaceous glue historically called “barnacle cement” to achieve durable underwater attachment. Since the discovery of methods to effectively solubilize the cured cement, a complex array of cement proteins (CPs) have been isolated from the acorn barnacle *Megabalanus rosa* and six of them have been thoroughly characterized. Based on the distinct amino acid compositions [3, 16], these CPs were classified into the following four categories: (1) Six amino acid-biased proteins, cp68k and cp19k [17], which have significantly high proportions of serine (Ser), threonine (Thr), glycine (Gly), alanine (Ala), lysine (Lys) and valine (Val); (2) Cysteine (Cys) and charged amino acids enriched protein, cp20k [18, 19]. It was found that the cp20k in *M*. *rosa* (Mrcp20k) contains as much as 17.5% Cys, and that the regular alignment of these Cys residues results in six degenerated repeats [18]; (3) Hydrophobic CPs, the major protein components in the cement, including cp100k [20] and cp52k [21]. The identification of *β*-sheet secondary structures in cp100k [20], as well as the observation of amyloid-like fibers self-assembled by peptide segments from cp52k [22], collectively indicates the crucial role of hydrophobic interactions in bulk cohesion of barnacle cement; (4) cp16k, which shares 47% identify to the lysozyme-P of *Drosophila melanogaster* [3]. Since no post-translational modifications have been identified in any barnacle adhesive proteins apart from glycosylation in cp52k [21], it is believed that barnacle possesses a novel underwater adhesion system that is different from mussel’s and tubeworm’s [23, 24]. In a hypothetical model of barnacle underwater attachment proposed by a combination of both structural and functional analysis, cp20k, cp19k and cp68k were suggested to play a surface adhesion role while cp52k and cp100k play a bulk cohesion role [3]. However, the complicated intermolecular interactions involved in surface adhesion and bulk cohesion of barnacle cement in this model remain largely unknown.

Besides, extensive research has been performed to investigate natural barnacle cement using scanning electron microscopy with energy dispersive spectrometry (SEM-EDS) [25–27], atomic force microscopy (AFM) [25, 28], Fourier transform infrared (FTIR) spectroscopy [25–29], etc., aiming to characterize its nanoscale morphologies and mechanics, chemical composition and secondary structures. AFM single molecular force study on the cement of *Balanus amphitrite* revealed regular sawtooth-like force-extension curves, with two average separation lengths of 35 ± 8 nm and 56 ± 9 nm, which were attributed to the hydrophobic interactions between segmented hydrophobic blocks in cp100k [25]. It thus suggested that the self-assembly of barnacle cement was possibly regulated by hydrophobic interactions of the bulk CPs. Combining nanoscale morphological observations with specific chemical staining confirmed the presence of amyloid fibers in *B*. *amphitrite* cement [25, 28], which was consistent with the identification of *β*-sheet secondary structures in cp100k and cp52k [20, 22], testifying the assumption that hydrophobic interactions played a key role in bulk cohesion of barnacle cement. It was also discovered that barnacle cement displayed differing morphologies on substrates with various chemistry, surface energy and elastic module [30, 31], especially between the easy-to-attach and non-stick surfaces. These findings provide new insights to elucidate the antifouling mechanisms of non-stick surfaces and instruct the design of novel non-fouling materials.

In comparison, mussel underwater adhesion has already been extensively researched and better understood. The pioneering studies on mussel wet adhesion have successfully established a universal platform, which lays the experimental or technical basis for working on other underwater adhesion systems [9]. The post-translationally modified tyrosine, 3,4-dihydroxyphenylalanine (DOPA) in mussel foot proteins (MFPs) acts essential roles by mediating various interactions, including hydrogen bonding, coordination with metals and metal oxides, as well as crosslinking after oxidized to dopaquinone [32–35]. To imitate the unique wet adhesion ability of MFPs, lots of attempts have been made to develop DOPA inspired materials [36–39]. For better design of novel mussel-inspired biomaterials, interactions between individual MFPs and various substrates have been widely investigated using a surface forces apparatus (SFA) in recent years [40, 41]. In parallel, the bulk adhesion strengths of different recombinant MFPs have also been fully examined using a shear strength test [42–45]. However, no reports to date have explored the adhesion strengths of barnacle CPs. To obtain a better understanding of barnacle underwater adhesion, it is necessary to investigate the adhesion strengths of barnacle CPs, especially those having a role in surface adhesion. Therefore, in this study we cloned and expressed the *cp19k* homologous gene from *Balanus albicostatus* (*Balcp19k*) in *Escherichia coli*, since it encodes a protein (Balcp19k) showing a remarkably distinct isoelectric point and relatively low homology compared to Mrcp19k (Table 1) [17], in order to verify and quantitatively evaluate its adhesion ability.

**Table 1:** A brief comparison of the physical characteristics of the three cp19k homologues.

	MWs	pIs	GenBank ID	Homology
**Mrcp19k**	17004.1	5.9	AB242294.1	100%
**Balcp19k**	17345.1	10.6	AB242295.1	54.34%
**Bicp19k** ^a^	16850.6	10.5	AB242296.1	51.45%

The molecular weights (MWs) and isoelectric points (pIs) of these three cp19k homologues were predicted with DNAman 6.0 based on their amino acid sequences. The homology was calculated according to the ClustalW2 alignment results of the three cp19k proteins.

^a^The cp19k homologue in *Balanus improvisus*.

Herein, we report the discovery of a super adhesive aggregation formed by the recombinant Balcp19k hybrid protein, Trx-Balcp19k, which was fused with an additional 18 kDa N-terminus encoded by pET-32a(+) plasmid. To evaluate its adhesion ability qualitatively and quantitatively, we first performed surface coating analysis and shear strength tests. We also experimentally analyzed its formation conditions. The biochemical composition was then examined to characterize its protein, total carbohydrate, and total lipid content. Based on these assays, we proposed a hypothetical mechanism for the formation of the adhesive protein aggregation.

## Materials and Methods

### Sample collection

The *B*. *albicostatus* samples were collected in February 2013 from the coast of the Chinese Yellow Sea in Qingdao, Shandong. The species is neither endangered nor protected. The collection location is a public region and we declare no economical conflicts. The barnacles that gregariously attached to rocks were carefully removed to ensure that their shells were intact to avoid any contamination of the inner soft tissues.

### Gene cloning and vector construction

**Fig 1:**
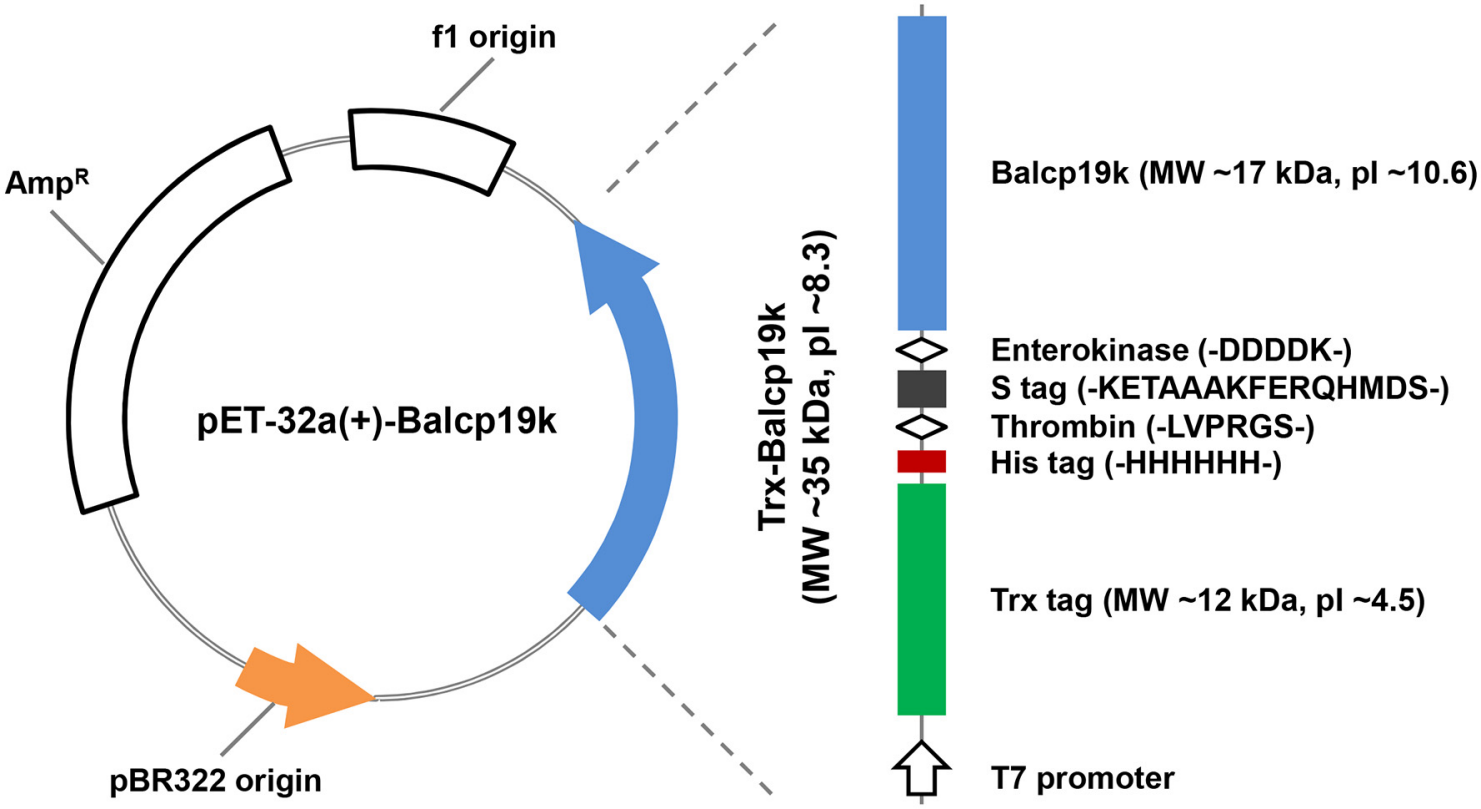
Structural schematic of Trx-Balcp19k. The Trx-Balcp19k is a hybrid protein with C-terminal Balcp19k and N-terminal Trx tag, His tag and S tag, as well as thrombin and enterokinase recognition sites.

The collected *B*. *albicostatus* samples were thoroughly rinsed with distilled water, and the barnacles with basal diameters of 5–10 mm were selected to isolate soft tissues for total RNA extraction. Total RNA was separated using TRIzol Reagent (Life technologies, CA, USA) and the integrity was evaluated by gel electrophoresis. The first strand complementary DNA (cDNA) synthesis was carried out using a FastQuant RT kit (with gDNase) (Tiangen, Beijing, China), with total RNA templates and oligo (dT)_20_ primers based on the standard protocol. To amplify the mature coding sequences (CDS) of *Balcp19k* (GenBank, AB242295.1), a pair of gene specific primers was designed as follows: forward primer *Balcp19k* F, 5’-GGAATTCGTGCCCCCACCGTGCGACCTCA-3’ and reverse primer *Balcp19k* R, 5’-ACGCGTCGACTCAGAGTCCCTTCAACTCGAGCT-3’, of which the underlined sequences were added as restriction enzyme recognition sites of *Eco*RI and *Sal*I, respectively. The *Balcp19k* CDS was amplified with high-fidelity Prime STAR HS DNA Polymerase (Takara, Dalian, China) via a touch-down polymerase chain reaction (PCR) procedure: preheating at 95°C for 1 min followed by 20 cycles of denaturing at 95°C for 30 s, annealing at temperatures decreasing 0.5°C per cycle from 70°C to 60°C for 30 s, and elongation at 72°C for 1 min. This was followed by an additional 20 cycles consisting of 95°C for 30 s, 60°C for 30 s, and 72°C for 1 min. The specifically amplified *Balcp19k* CDS was subjected to an A-tailing reaction with the DNA A-Tailing kit (Takara, Dalian, China) so that it could be subcloned into T-Vector pMD19 (simple) (Takara, Dalian, China) for sequencing. For vector construction, the *Balcp19k* CDS was digested from the recombinant T-Vector pMD19 (simple) with *Eco*RI and *Sal*I, and then inserted into the prokaryotic expression vector pET-32a(+) (EMD Chemicals, CA, USA). As shown in Fig 1, the Trx-Balcp19k fusion protein encoded by recombinant pET-32a(+)-Balcp19k plasmid is comprised of the C-terminal Balcp19k and an additional N-terminal fragment, which contains a thioredoxin (Trx) tag to promote intramolecular disulfide bonds formation, a His tag to allow simple purification of recombinant proteins through affinity chromatography, an S tag, as well as thrombin and enterokinase recognition sites to remove the tags. The recombinant Balcp19k fusion protein was named “Trx-Balcp19k” for brevity since Trx is the largest tag at the N-terminus. Finally, the constructed recombinant plasmids were sequenced to verify the absence of frameshift mutations.

### Recombinant protein expression and purification

For recombinant protein expression, the constructed plasmids were first transformed into *Escherichia coli* host strain BL21 (DE3) (Tiangen, Beijing, China). The transformed single bacterial colony was cultured in 25 ml Luria-Bertani (LB) liquid medium containing 50 μg/ml of ampicillin at 37°C with a shake speed of 220 revolutions per minute (rpm) overnight. Next, 20 ml of the bacterial suspensions were added into 2 L fresh LB liquid medium containing 50 μg/ml ampicillin at a ratio of 1/100 (v/v) and the cultures were incubated at 37°C with shaking at 220 rpm. The bacterial growth was monitored by optic density at 600 nm (OD_600_) and once it reached 0.6–0.8, recombinant protein expression was induced by the addition of 1 mM isopropyl-β-D-thiogalactopyranoside (IPTG). The cultures were induced at 37°C for an additional 5 h. The bacterial cells were harvested by centrifugation at 6000 rpm and 4°C for 10 min and stored at -80°C.

The Trx-Balcp19k was purified by affinity chromatography using Ni-NTA super flow resin (Qiagen, Hilden, Germany) according to the manufacturer’s instructions. Briefly, the harvested cell pellets were rinsed with precooled 10 mM phosphate buffered saline (PBS, pH 7.4) and resuspended in 80 ml binding buffer (50 mM Na_2_HPO_4_, 300 mM NaCl and 10 mM imidazole, pH 8.0) at a 25-fold concentration. To prepare soluble protein fractions, the bacterial suspensions were digested by lysozyme (Sangon, Shanghai, China) at a final concentration of 30–50 KU per gram of cell pellets at 30°C for 15 min, followed by 40 cycles of sonication at 17 watts for 10 s each. The lysates were then centrifuged at 14,000 rpm and 4°C for 30 min, and the resulting supernatant was coincubated with prewashed Ni-NTA super flow resins by shaking at 4°C for 1 h. The mixture was then loaded into dispensable plastic columns for chromatography. Non-specific absorbed proteins were rinsed with washing buffer (pH 8.0) containing 50 mM Na_2_HPO_4_, 300 mM NaCl and 50 mM imidazole, while the specific recombinant proteins were eluted with 24 ml eluting buffer (50 mM Na_2_HPO_4_, 300 mM NaCl and 250 mM imidazole, pH 8.0). The purity and production of isolated Trx-Balcp19k were estimated by sodium dodecyl sulfate-polyacrylamide gel electrophoresis (SDS-PAGE).

### Formation of adhesive aggregation

**Fig 2:**
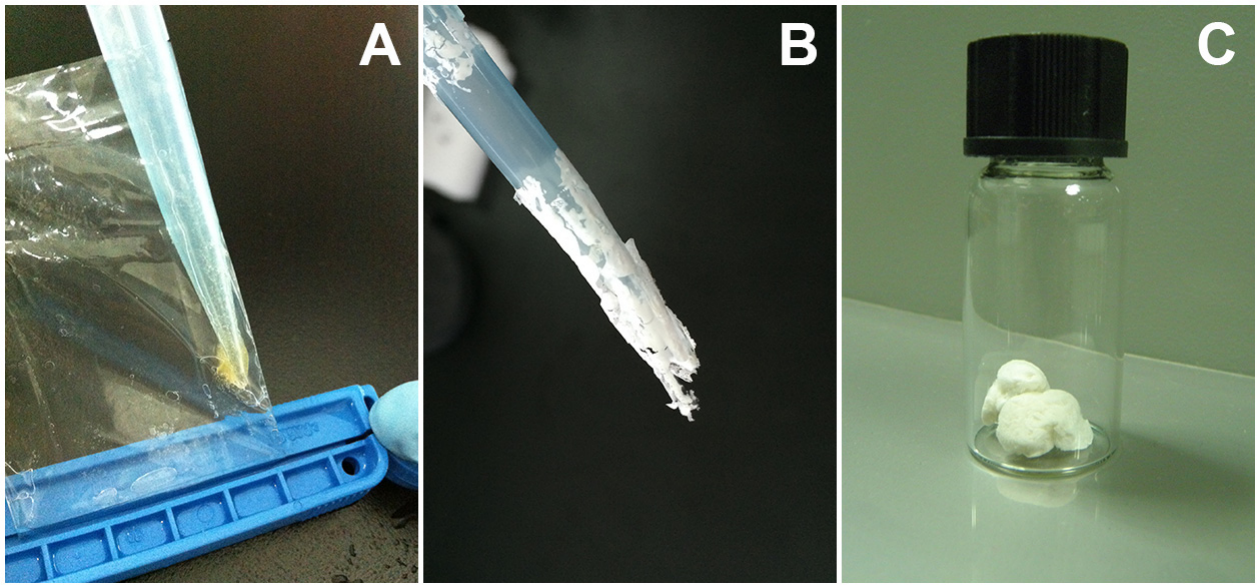
Adhesive aggregation formed by Trx-Balcp19k when dialyzed against Milli-Q water. (A) Sticky aggregation was generated after Trx-Balcp19k eluate was dialyzed against pure water, which was termed “Trx-Balcp19k gel” for simple description. (B) The slices of lyophilized Trx-Balcp19k gel. (C) The disaggregated Trx-Balcp19k exhibited a foam-like morphology when freeze dried, and therefore it was termed “Trx-Balcp19k foam”.

In order to remove the high concentration of imidazole in the Trx-Balcp19k elute, it was dialyzed against Milli-Q water at 4°C overnight. Interestingly, we observed a trace amount of sticky gel-like aggregation absorbed on the dialysis membrane (Spectrum, USA). For convenient description, we named this material “Trx-Balcp19k gel” although it was not strictly gel *per se*. The sticky Trx-Balcp19k gel was first discovered when we were transferring the dialysis products because it easily blocked the pipette tips (Fig 2). In addition, we found that it remained clear after the Trx-Balcp19k eluate was dialyzed against 10 mM PBS (pH 7.4) and phosphate buffer (pH 8.0) containing 50 mM Na_2_HPO_4_ and 50 mM NaCl. These results indicated that the formation of Trx-Balcp19k gel may be pH- and/or ionic strength-dependent. Therefore, to further investigate their effects on the formation of the adhesive aggregation, the Trx-Balcp19k eluate was dialyzed against 5% (v/v) acetic acid (pH ~2.5) and 0.1 M NaCl, respectively.

### Evaluation of the adhesion ability of Trx-Balcp19k gel

Surface coating analysis [37] was used to qualitatively assess the adsorption ability of Trx-Balcp19k gel. The hydrophobic tissue culture polystyrene plates (Corning, NY, USA) were directly used without any pretreatments, while the hydrophilic glass slides were bathed in 5% (v/v) hydrochloric acid overnight, rinsed with Milli-Q water, and dried in air. To perform surface coating analysis, 10 μl of 1.33 mg/ml Trx-Balcp19k gel solution dissolved in 5% (v/v) acetic acid was added onto the two substrates. After fully adhering to the substrates, the Trx-Balcp19k gel on the two surfaces was rinsed with Milli-Q water and the absorbed proteins were visualized by Coomassie brilliant blue staining. Cell-Tak (Discovery Labware, MA, USA), which is a mixture of extracted natural MFPs secreted by *Mytilus edulis* in 5% (v/v) acetic acid, was used as positive control, whereas lysozyme (MP Biomedicals, OH, USA) and bovine serum album (BSA) (Affymetrix, OH, USA) were used as negative controls. The experiments were performed with at least three independent replicates.

**Fig 3:**
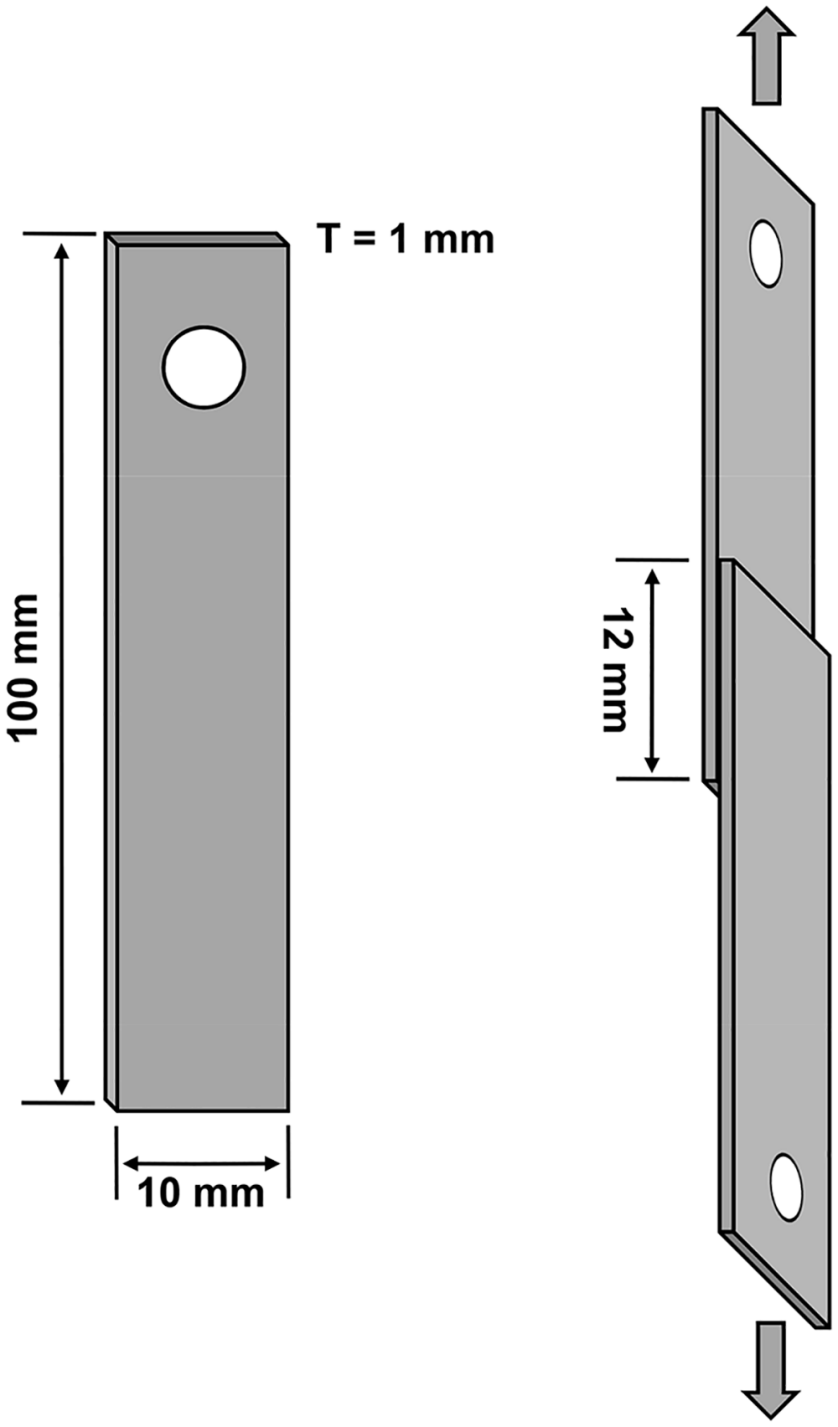
Schematic illustration of the single-lap-joint shear strength test. Aluminum plate with a thickness of ~1 mm was cut into 100 mm × 10 mm sheets. A small hole was drilled at one end of the aluminum sheet for convenient fixation. During the test, two adhesively-bonded aluminum adherents with an overlapping area of 12 mm × 10 mm were torn apart in the directions indicated by the arrows.

The single-lap-joint shear strength test was adapted from the description of Cha et al. [42] to quantitatively characterize the adhesion strength of Trx-Balcp19k gel. First, an aluminum plate with a thickness of ~1 mm was cut into 100 mm × 10 mm sheets with a small hole at the end for convenient fixation (Fig 3). These aluminum sheets were sequentially treated by soaking in 5% (w/v) NaOH for 5 min, rinsing with distilled water, soaking in 30% (v/v) HNO_3_ for 1 min, rinsing with distilled water again, and subsequently air dried. Adhesively bonded samples were prepared as follows: about 5 mg lyophilized Trx-Balcp19k gel were weighed and placed on the end of one aluminum sheet, 10 μl of Milli-Q water was then added, and another aluminum sheet was placed over the samples with an overlapping area of 12 mm x 10 mm (Fig 3). To ensure that the samples were evenly distributed, the two aluminum sheets were pressed together several times. Before the tests, the samples were fixed with clips and allowed to cure thoroughly at 37°C. The shear strength tests were performed on a universal tensile test machine with multiple mechanical sensors at a crosshead speed of 0.5 mm/min. The adhesive shear strengths were calculated by dividing the maximal load (N) with the overlapping area (m^2^), based on the recorded load-distance curves. BSA and lysozyme were used as negative controls while commercial polyvinyl acetate (PVA) craft glue (Mont Marte, Australia) and UHU all purpose adhesive (UHU GmbH & Co. KG, Germany) were used as positive controls. All tests were conducted with at least three repetitive trials. The collected data were shown as mean ± standard deviation.

### Biochemical composition analysis of Trx-Balcp19k gel

We performed a biochemical composition assay of Trx-Balcp19k gel to characterize its protein, total carbohydrate, and total lipid content. To determine the protein components, we first conducted SDS-PAGE of the Trx-Balcp19k gel under both reducing and non-reducing conditions. Total carbohydrate quantification of the Trx-Balcp19k gel was conducted using the phenol-sulfuric acid method with D-glucose (Sigma, MO, USA) as a standard [46]. Briefly, 1 ml of 2.5 mg/ml Trx-Balcp19k gel solution in 5% (v/v) acetic acid was mixed with the reaction buffer, which was composed of 1 ml 5% (w/v) phenol (Sigma, MO, USA) and 2.5 ml 98% concentrated sulfuric acid. After incubation in darkness for 15 min, its optic absorbance at 490 nm (A490) was measured using UV-2550 spectrophotometer (Shimadzu, Japan). The total lipid content of Trx-Balcp19k gel was also examined employing the sulfo-phospho-vanillin (SPV) method with triolein (Sigma, MO, USA) as a standard [47, 48]. The 20 mg/ml triolein storage solution was prepared by dissolving 20 mg triolein in 1 ml absolute ethanol. To prepare the phospho-vanillin reagent, 350 ml vanillin solution (6 mg/ml in ddH_2_O), 50 ml ddH_2_O, and 600 ml concentrated phosphoric acid were thoroughly mixed and then stored at room temperature. For determination of the total lipid content in Trx-Balcp19k gel, 20 μl of absolute ethanol and 200 μl of concentrated sulfuric acid were sequentially added to 2.5 mg lyophilized Trx-Balcp19k gel and incubated in boiling water for 10 min. After cooling, it was mixed with 5 ml phospho-vanillin reagent and reacted at 37°C for 15 min. Finally, the optic absorbance was measured at 540 nm (A540).

## Results

### Gene cloning and vector construction

**Fig 4:**
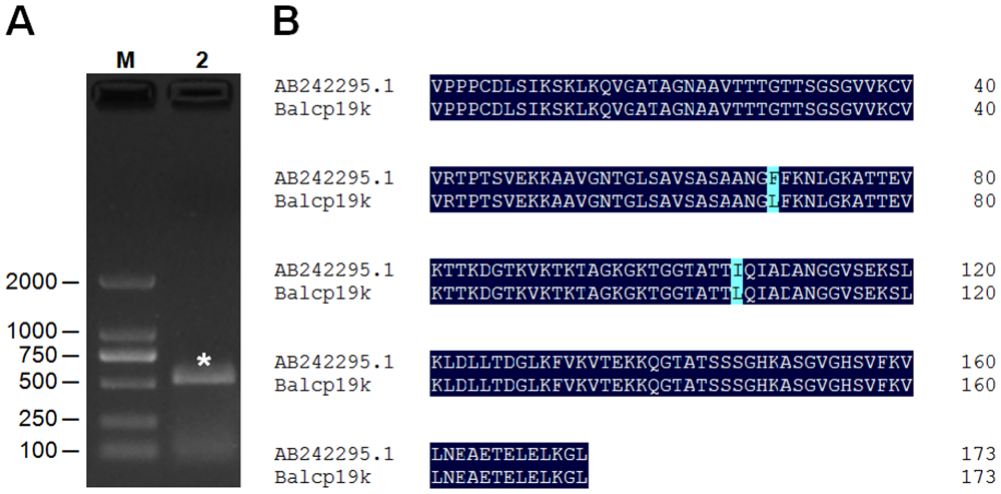
Gene cloning results of *Balcp19k*. (A) Gel electrophoresis of RT-PCR amplified *Balcp19k* CDS. Lane M: DL2000 DNA Marker (Takara, Dalian, China), Lane 2: the amplified *Balcp19k* CDS as indicated by the pentacle. (B) Amino acid sequence alignment of Balcp19k with DNAman 6.0. The Phe^69^ (F) and Ile^106^ (I) of AB242295.1 (GenBank ID) were both substituted with Leu (L) in the Balcp19k amplified in this study.

As previously reported by Urushida et al. [17], the mature coding sequence of *Balcp19k* possesses 522 base pairs that encode a 173 amino acid protein. Based on the specific band observed by gel electrophoresis (Fig 4A), we concluded that the *Balcp19k* CDS was successfully amplified. Unexpectedly, sequencing revealed that the amplified *Balcp19k* CDS in our study showed two amino acid differences (L69F, L106I) compared to the sequence submitted to GenBank (AB242295.1) (Fig 4B). These differences can be attributed to the polymorphisms present in cp19ks from different *B*. *albicostatus* subspecies, since the barnacles we collected were obtained from a different region than those from the other study. Therefore, we kept the two distinct amino acids. For expression vector construction, the amplified *Balcp19k* CDS was inserted into pET-32a(+) and the corresponding recombinant plasmid was designated as pET-32a(+)-Balcp19k. As illustrated in Fig 1, the recombinant Trx-Balcp19k fusion protein encoded by pET-32a(+)-Balcp19k plasmid contains an ~18 kDa fragment consisting of a Trx tag, a His tag and an S tag, as well as thrombin and enterokinase sites at the N-terminus, and Balcp19k at the C-terminus.

### Recombinant protein expression and purification

**Fig 5:**
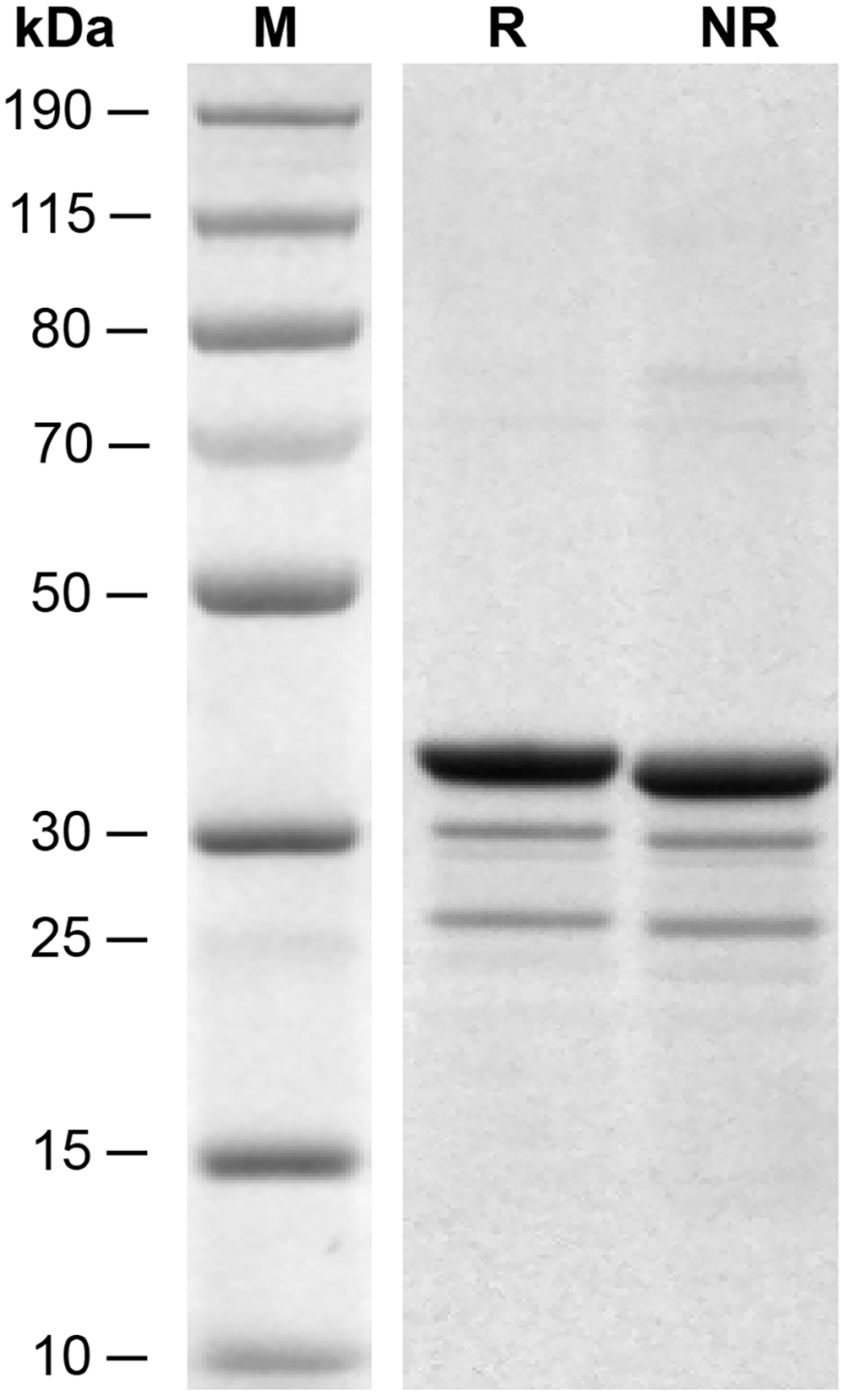
SDS-PAGE of purified Trx-Balcp19k by affinity chromatography. Lane M is the PageRuler Plus Prestained Protein Ladder (Pierce Biotechnology, IL, USA). The R and NR are abbreviations for reducing and non-reducing SDS-PAGE, respectively. The non-specific bands may be due to degradation and/or pre-termination of ORF translation. Moreover, the different migration patterns of Trx-Balcp19k between reducing and non-reducing conditions suggest that the two Cys residues in Balcp19k had possibly formed an intramolecular disulfide bond with the aid of Trx tag.

Due to the fused N-terminal tags and sites, the Trx-Balcp19k contains 340 amino acids with a deduced MW of ~35321.0 Da and a calculated pI of ~8.3, according to the prediction of DNAman 6.0. The Trx-Balcp19k was expressed in soluble form in *E*. *coli* and purified by affinity chromatography. As shown in Fig 5, the apparent MW of purified Trx-Balcp19k judged from SDS-PAGE was in accordance with its deduced MW, indicating that the Trx-Balcp19k was successfully purified. We produced and purified 20–50 mg of Trx-Balcp19k from a 2-L bacterial culture. However, the non-target bands in SDS-PAGE suggested that it incurred slight degradation and/or pre-termination of open reading frame (ORF) translation (Fig 5). A Trx tag was added at the N-terminus of Balcp19k to promote the formation of intramolecular disulfide bonds, because it was thought to be important for maintaining the natural conformation of cp19k [17]. We inferred that in the majority of Trx-Balcp19k the four Cys residues had probably formed two intramolecular disulfide bonds, because only a small number of multimers were generated even when Trx-Balcp19k eluate was exposed in air overnight. It was consistent with the different migration patterns present in the reducing and non-reducing SDS-PAGE (Fig 5). Furthermore, it could be supposed that the two Cys residues in Balcp19k and the other two in Trx tag respectively crosslinked to form one intramolecular disulfide bond each, based on their spatial locations in Trx-Balcp19k.

### Formation conditions of Trx-Balcp19k gel

As mentioned before, we unintentionally discovered that Trx-Balcp19k formed a small amount of adhesive aggregation (Trx-Balcp19k gel), when the eluate was dialyzed against pure water to remove the high concentration of imidazole. Interestingly, the Trx-Balcp19k gel appeared very sticky, and to the best of our knowledge frog glue is the only biomaterial that is known to possess this unique property [49]. Based on our observations, the production of Trx-Balcp19k gel was influenced by the concentration of Trx-Balcp19k eluate and dialysis time. It was estimated that 10–20 mg Trx-Balcp19k gel could be collected from a 2-L bacterial culture. We evaluated the impact of pH and ionic strength on the formation of Trx-Balcp19k gel by dialyzing the eluate against 5% (v/v) acetic acid (pH ~2.5) and 0.1 M NaCl respectively, since the gel could not be generated through dialysis to 10 mM PBS (pH 7.4) and phosphate buffer (50 mM Na_2_HPO_4_ and 50 mM NaCl, pH 8.0). We found that the Trx-Balcp19k eluate remained clear after dialysis against 0.1 M NaCl, but formed some non-sticky pellets composed of non-target proteins as revealed by SDS-PAGE after dialysis against 5% (v/v) acetic acid. Overall, these results suggest that the Trx-Balcp19k gel formation is dependent on pH and ionic strength. Therefore, electrostatic interactions play an important role.

### Adhesion ability of Trx-Balcp19k gel

**Fig 6:**
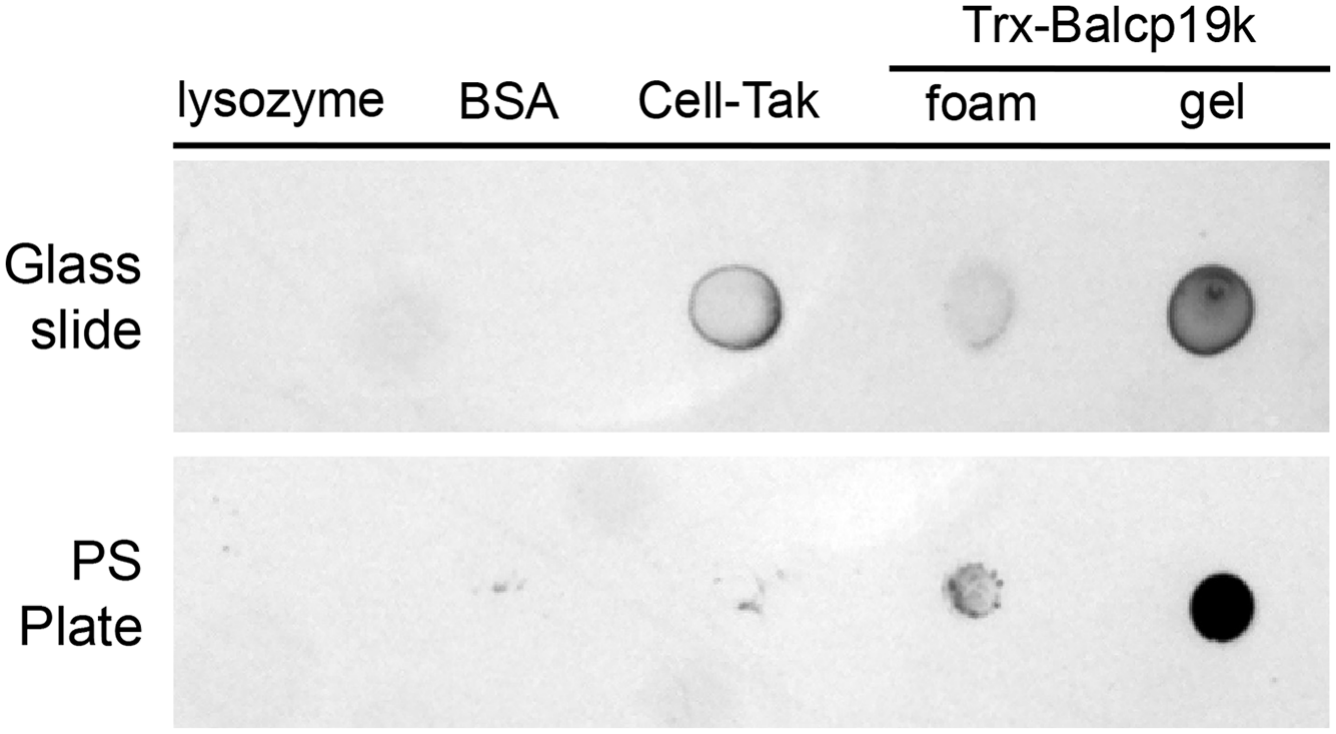
Surface coating analysis of Trx-Balcp19k gel on hydrophilic glass slides and hydrophobic polystyrene (PS) plates.

For qualitatively assessing the surface adsorption ability of Trx-Balcp19k gel, surface coating analysis was conducted on two of most commonly used bio-related surfaces: hydrophilic glass slides and hydrophobic polystyrene tissue culture plates. As shown in Fig 6, the Trx-Balcp19k gel clearly exhibited high adsorption on both of the two substrates, whereas almost all absorbed lysozyme and BSA were washed away, as expected. The positive control Cell-Tak displayed higher adsorption on hydrophilic glass slides, but Trx-Balcp19k gel showed stronger adsorption on hydrophobic polystyrene substrates, which was probably due to their differences in hydrophobicity. Since polystyrene is the most often used *in vitro* cell culture substrate, the Trx-Balcp19k gel can thus be potentially used as an adhesive layer to regulate cell behavior [50, 51]. The adhesion ability of disaggregated Trx-Balcp19k (Trx-Balcp19k foam) was also qualitatively evaluated. However, the foam demonstrated much lower surface adsorption compared to Trx-Balcp19k gel on both surfaces.

**Fig 7:**
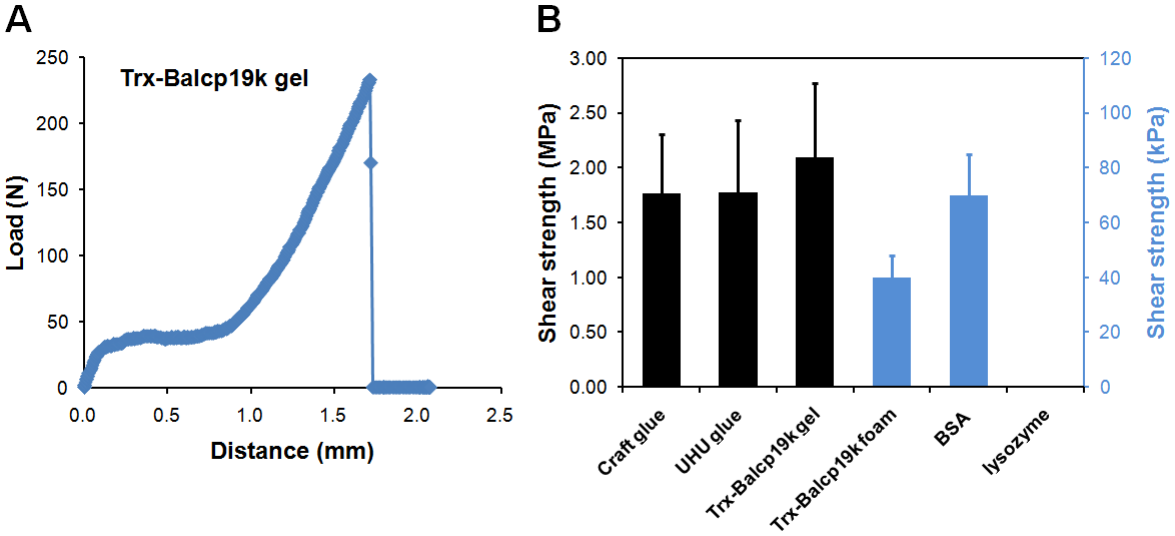
Adhesion strength of Trx-Balcp19k gel measured using a single-lap-joint shear strength test. (A) Representative load-distance profile of Trx-Balcp19k gel. (B) Adhesive shear strengths of several protein samples. The aggregated Trx-Balcp19k (Trx-Balcp19k gel) showed a greater adhesion strength (2.10 ± 0.67 MPa) compared to PVA craft glue (1.77 ± 0.54 MPa) and UHU glue (1.78 ± 0.65 MPa), while the disaggregated Trx-Balcp19k (Trx-Balcp19k foam) had an extremely low adhesion strength (40 ± 8 kPa), which was even lower than that of BSA (70 ± 15 kPa).

We also used a single-lap-joint shear strength test to quantitatively measure the bulk adhesion strength of Trx-Balcp19k gel. The collected representative load-distance curve of Trx-Balcp19k gel was shown in Fig 7A. We found that the Trx-Balcp19k gel had an adhesive shear strength of 2.10 ± 0.67 MPa, which was higher than those of commercial PVA craft glue (1.77 ± 0.54 MPa) and UHU glue (1.78 ± 0.65 MPa) (Fig 7B). On the other hand, the disaggregated Trx-Balcp19k (Trx-Balcp19k foam) showed very low adhesion strength of 40 ± 8 kPa, which was even weaker than that of the negative control BSA (70 ± 15 kPa) (Fig 7B). The significantly different adhesion strengths between Trx-Balcp19k gel and Trx-Balcp19k foam suggested that the adhesion ability of Trx-Balcp19k was dramatically strengthened by aggregation. Additionally, the aluminum sheets adhesively bonded by the control lysozyme were easily torn apart by gravity once the clip was removed, indicating its extremely low adhesion strength. The bulk adhesion strengths of different adhesive proteins and coacervates, including recombinant MFPs and frog glue, are summarized in Table 2. However, it is difficult to make exact comparisons of these properties due to differences in experimental conditions.

**Table 2:** Summaries of the adhesion strengths of several adhesive proteins and coacervates.

Adhesive proteins and coacervates	Adhesive shear strengths (MPa)	References
**fp-3**	0.57 ± 0.21 (unmodified)	[45]
	0.94 ± 0.33 (modified, uncrosslinked)	
	2.28 ± 0.41 (modified, crosslinked)	
**fp-5**	0.69 ± 0.17 (modified, uncrosslinked)	[45]
	1.76 ± 0.45 (modified, crosslinked)	
**fp-131**	1.64 ± 0.22 (modified, uncrosslinked)	[45]
	0.71 ± 0.15 (modified, crosslinked)	
**fp-151**	1.31 ± 0.11 (modified, uncrosslinked)	[45]
	0.88 ± 1.01 (modified, crosslinked)	
**fp-131/HA**	4.00 ± 0.53 (modified)	[43]
**fp-151/HA**	3.17 ± 0.51 (modified)	[43]
**frog glue**	1.70 ± 0.30	[49]

The “modified” and “unmodified” refer to recombinant MFPs with and without DOPA moieties, respectively. The “crosslinked” notation refers to recombinant MFPs that were crosslinked through DOPA oxidation and the “uncrosslinked” notation refers to recombinant MFPs that were not crosslinked. The adhesive shear strength of Trx-Balcp19k gel (2.10 ± 0.67 MPa) is comparable to those of recombinant MFPs and frog glue, but weaker than those of recombinant MFPs/hyaluronic acid (HA) coacervates.

### Biochemical composition of Trx-Balcp19k gel

**Fig 8:**
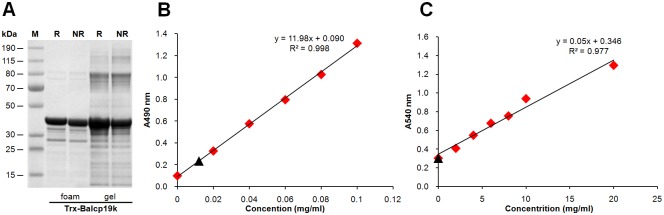
Biochemical composition of the Trx-Balcp19k gel. The SDS-PAGE analysis (A) indicated that the Trx-Balcp19k gel was actually non-covalently aggregated soluble Trx-Balcp19k based on the identical protein bands observed between Trx-Balcp19k gel and Trx-Balcp19k foam. The Trx-Balcp19k gel is proteinaceous, since only trace amounts of carbohydrates (B) and no lipids (C) were detected.

It was amazing that the Trx-Balcp19k gel had adhesive shear strength comparable to those of referential commercial glues (Fig 7B). To the best of our knowledge, no purified protein to date has exhibited such strong adhesion ability. It is rational to question whether the Trx-Balcp19k gel contains carbohydrates and/or lipids, since the dialysis membrane is made of cellulose that may be incorporated into the aggregation. Moreover, proteins and lipids have been reported to synergistically interact in natural barnacle adhesive [52]. Therefore, we assessed the biochemical composition of Trx-Balcp19k gel. The SDS-PAGE result (Fig 8A) revealed that the sticky aggregation was mainly composed of Trx-Balcp19k monomers as well as some non-specific protein fractions. It could be concluded that intermolecular disulfide bonds were not responsible for the aggregation of Trx-Balcp19k, which was consistent with the deduction that in the majority of Trx-Balcp19k all the four Cys residues were employed to form intramolecular disulfide bonds. In addition, given the similar but stronger bands of the gel than the foam, we surmised that Trx-Balcp19k gel was actually non-covalently aggregated Trx-Balcp19k foam. The total carbohydrate content of Trx-Balcp19k gel was determined to be approximately 0.48% (w/w, about 12 μg total carbohydrate in 2.5 mg Trx-Balcp19k gel) using the phenol-sulfuric acid method (Fig 8B), while no lipids were detected using the SPV method (Fig 8C). These results suggested that the Trx-Balcp19k gel was proteinaceous and its formation relied on self-aggregation of Trx-Balcp19k.

## Discussion

Barnacle underwater attachment represents a wet adhesion system that relies on the special amino acid composition, secondary and ternary structures, and crosslinks of cement proteins, rather than DOPA moieties which are widely employed in mussel foot proteins and tubeworm cement proteins. In order to gain an understanding about barnacle underwater adhesion, it is necessary to investigate the adhesion abilities of barnacle cement proteins. In this study we cloned and expressed the *cp19k* homologous gene of *B*. *albicostatus* in *E*. *coli*. It should be noted that our amplified *Balcp19k* CDS showed two amino acid differences (L69F, L106I) compared to the sequence in GenBank (AB242295.1) (Fig 4B), possibly due to the polymorphisms in cp19ks from different *B*. *albicostatus* subspecies. The recombinant Trx-Balcp19k, which was combined with an additional 18 kDa N-terminus consisting of a Trx tag, a His tag, an S tag, as well as thrombin and enterokinase sites (Fig 1), was one-step purified via affinity chromatography. The high production yield allowed us to directly measure its macroscopic adhesion strength. The bulk shear strength test revealed an adhesion strength of 40 ± 8 kPa for disaggregated Trx-Balcp19k (Trx-Balcp19k foam), which was next to the reported value (90 ± 40 kPa) of barnacle cement secretion 1 (BCS1) of *B*. *amphitrite* [53]. Since barnacles attach themselves to underwater substrates so tenaciously that any attempts to dislodge them only lead to breaks in the peripheral shells, there seems to be a paradox between the relatively weak adhesion of disaggregated Trx-Balcp19k and the strong adhesion of natural barnacle cement, but natural barnacle underwater adhesive is actually a multi-protein complex. What’s more, it was worthy of being noted that our obtained adhesion strength was property of Trx-Balcp19k fusion protein, which cannot represent the real adhesion ability of native Balcp19k, due to the combination of an additional 18 kDa N-terminus. Therefore, in this study we were not able to accurately evaluate the adhesion strength of Balcp19k without removing these combined N-terminal tags.

What’s really impressed us was that the eluted Trx-Balcp19k in affinity chromatography aggregated into a super sticky, gel-like substance when dialyzed against Milli-Q water, which was named Trx-Balcp19k gel. It is common for proteins to aggregate and precipitate when dialyzed against pure water, since solubility depends to a large extent on the ionic strength of the solution. However, we believe that the formation of Trx-Balcp19k gel is quite intriguing, because to the best of our knowledge, only frog glue forms such a strong adhesive aggregation when dialyzed against pure water [49]. The macroscopic adhesion strength of Trx-Balcp19k gel was measured to be 2.10 ± 0.67 MPa, which was comparable to those of frog glue, crosslinked recombinant mfp-3 and mfp-5, and commercial PVA craft glue and UHU glue. Although the adhesion strengths of Trx-Balcp19k gel and crosslinked recombinant MFPs were comparable, the dependence of MFPs on post-translational modifications poses challenges in producing functional recombinant forms of the proteins [54, 55]. From this prospective, the Trx-Balcp19k gel seems more promising. Furthermore, the remarkably distinct adhesion strength between disaggregated (Trx-Balcp19k foam) and aggregated Trx-Balcp19k (Trx-Balcp19k gel) suggests that the adhesion ability of Trx-Balcp19k can be greatly reinforced by aggregation.

To understand how the adhesive Trx-Balcp19k gel was formed, we conducted a series of characterization assays. First, the biochemical composition was investigated in order to characterize the protein, total carbohydrate, and total lipid content. As shown in Fig 8, SDS-PAGE revealed that the adhesive aggregation was mainly composed of Trx-Balcp19k monomers as well as a few non-target bacterial proteins. Moreover, it was observed that Trx-Balcp19k gel showed quite similar but stronger bands compared to Trx-Balcp19k foam. These results may suggest that the gel was non-covalently aggregated Trx-Balcp19k. No lipids and only trace amounts of carbohydrates were detected in Trx-Balcp19k gel using colorimetric methods, indicating the proteinaceous nature of the gel and self-aggregation property of Trx-Balcp19k. Since the adhesive aggregation only formed when Trx-Balcp19k eluate was dialyzed against pure water, it remained clear when dialyzed against 10 mM PBS (pH 7.4) and phosphate buffer (pH 8.0) containing 50 mM Na_2_HPO_4_ and 50 mM NaCl. Therefore, we next evaluated the effect of pH and ionic strength on its formation by dialyzing Trx-Balcp19k eluate against 5% (v/v) acetic acid (pH ~2.5) and 0.1 M NaCl, respectively. We discovered that some non-adhesive pellets formed when the eluate was dialyzed against 5% (v/v) acetic acid, but remained clear when dialyzed against 0.1 M NaCl, indicating that Trx-Balcp19k gel formation was pH- and ionic strength-dependent. Taken together these results suggested that electrostatic interactions play a vital role in the gel formation.

**Fig 9:**
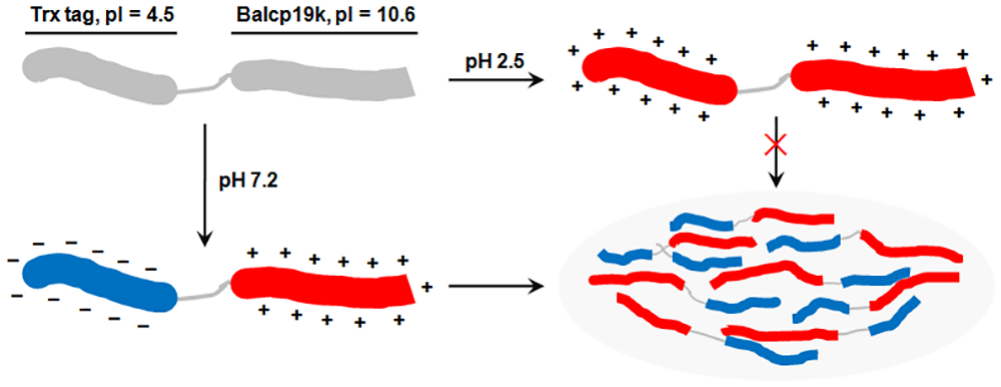
Schematic illustration of electrostatic interactions triggering self-aggregation of Trx-Balcp19k. The N-terminus of Trx-Balcp19k contains a Trx tag, which is rich in acidic amino acids and has a calculated pI of ~4.5, while the C-terminus is Balcp19k which contains numerous basic amino acids and has a theoretical pI of ~10.6. As a result, when Trx-Balcp19k eluate was dialyzed against solutions with low ionic strength and a pH between 4.5 and 10.6, such as at pH 7.2 which was the pH where the adhesive aggregation formed, Trx-Balcp19k had different net charge density at its N- (about -0.05/residue) and C-terminus (about +0.07/residue), thus leading to electrostatic attraction triggered self-aggregation. On the contrary, when it was dialyzed against solutions with low ionic strength and a pH higher than 10.6 or lower than 4.5, such as 5% (v/v) acetic acid (pH ~2.5), both ends of Trx-Balcp19k displayed the same net charges, resulting in strong electrostatic repulsion that prevented aggregation.

Based on the above analyses, we infer that the formation of adhesive Trx-Balcp19k gel is caused by self-aggregation of soluble Trx-Balcp19k, which is characteristic of Trx-Balcp19k hybrid protein and triggered by electrostatic interactions. Therefore, one question that arises is how do electrostatic interactions mediate Trx-Balcp19k gel formation? Through careful Trx-Balcp19k sequence analysis, we postulated that the extremely asymmetrical distribution of charged amino acids in Trx-Balcp19k may be responsible for the formation of the Trx-Balcp19k gel. As illustrated in Fig 9, the predicted pIs of the N-terminal Trx tag and the C-terminal Balcp19k was ~4.5 and ~10.6, respectively, based on DNAman 6.0 analysis. That was to say, the charged amino acids were unevenly distributed, with acidic residues concentrated at the N-terminus and basic residues clustered at the C-terminus. When Trx-Balcp19k eluate was dialyzed against solutions with low ionic strength and a pH between 4.5 and 10.6, for example, at pH 7.2 that was the pH where Trx-Balcp19k gel formed, the N-terminal Trx tag (MW ~12 kDa) had a charge density of about -0.05/residue while the C-terminal Balcp19k (MW ~17 kDa) had a charge density of about +0.07/residue. As a result, Trx-Balcp19k was gradually aggregated through electrostatic attraction and finally formed visible sticky gel-like material. In contrast, when Trx-Balcp19k eluate was dialyzed against solutions with low ionic strength and a pH where Trx tag and Balcp19k carry the same surface charges, such as 5% (v/v) acetic acid (pH ~2.5), its solubility was enhanced by electrostatic repulsion and no adhesive aggregation occurred. Dialysis against a buffer with high ionic strength also promoted solubility of Trx-Balcp19k through a salting-in effect. Similarly, electrostatic interactions induced coacervation was also hypothesized in the cement of sandcastle worm *Phragmatopoma californica* [4] and observed in Mfp-3S variant of *Mytilus californianus* [56].

It is attractive that the adhesion strength of Trx-Balcp19k can be greatly enhanced by self-aggregation, although the detailed mechanism is currently unclear. We suppose that the electrostatic interactions triggering self-aggregation is beneficial for Trx-Balcp19k to achieve high surface density and optimize its spatial alignment. Similarly, the amyloid-like fibers self-assembled by the recombinant MFPs, CsgA-Mfp3 and Mfp5-CsgA also demonstrate much higher adhesion strength than other recombinant MFPs, even natural MFPs [10]. These results indicate the possibility that the functions of biomolecules may be improved by ordered self-assembly. Based on these findings, we propose a strategy for designing peptide- or protein-based materials, that is, by combining self-assembly building blocks with functional biomolecules, and utilizing the ordered self-assembly of structural units to optimize the spatial conformation and enhance the functions of biomolecules.

## Conclusions

In this study, the *cp19k* homologous gene of *Balanus albicostatus* (*Balcp19k*) was successfully cloned and expressed in *E*. *coli*. Sequence analysis showed that the Phe^69^ and Ile^106^ of Balcp19k (GenBank No. AB242295.1) were both substituted by Leu in this amplified Balcp19k, which was likely due to polymorphisms of cp19ks from different subspecies. We found that Trx-Balcp19k self-aggregated into a gel-like substance named Trx-Balcp19k gel when dialyzed against pure water overnight. The Trx-Balcp19k gel is of particular interest because it had significantly high adhesion strength of 2.10 ± 0.67 MPa, which was much stronger than that of disaggregated Trx-Balcp19k (40 ± 8 kPa) and rivaled those of commercial PVA craft glue and UHU glue. The Trx-Balcp19k gel was found to be proteinaceous and mainly composed of Trx-Balcp19k monomers. Electrostatic interactions, rather than covalent crosslinking, triggered the self-aggregation of Trx-Balcp19k, which may be dependent on the unique asymmetrical distribution pattern of charged amino acids, since we discovered that acidic residues were concentrated at the N-terminus while basic residues were concentrated at the C-terminus. These findings not only demonstrate a promising, strong proteinaceous adhesive with potential application in various biomedical fields, but also provide new insight for the design of peptide- or protein-based biomaterials.
